# A target Capture Probe Set Useful for Deep- and Shallow-Level Phylogenetic Studies in Cactaceae

**DOI:** 10.3390/genes13040707

**Published:** 2022-04-17

**Authors:** Monique Romeiro-Brito, Milena Cardoso Telhe, Danilo Trabuco Amaral, Fernando Faria Franco, Evandro Marsola Moraes

**Affiliations:** 1 Departamento de Biologia, Centro de Ciências Humanas e Biológicas, Universidade Federal de São Carlos (UFSCar), Sorocaba 18052-780, SP, Brazil; romeiro.monique@gmail.com (M.R.-B.); milena.telhe@gmail.com (M.C.T.); danilo.trabuco@gmail.com (D.T.A.); franco@ufscar.br (F.F.F.); 2 Programa de Pós-graduação em Biologia Comparada, Faculdade de Filosofia, Ciências e Letras de Ribeirão Preto, Universidade de São Paulo (USP), Ribeirão Preto 14040-901, SP, Brazil

**Keywords:** phylogenomics, cacti, coalescent inference, rapid radiation, target capture sequencing

## Abstract

The molecular phylogenies of Cactaceae have enabled us to better understand their systematics, biogeography, and diversification ages. However, most of the phylogenetic relationships within Cactaceae major groups remain unclear, largely due to the lack of an appropriate set of molecular markers to resolve its contentious relationships. Here, we explored the genome and transcriptome assemblies available for Cactaceae and identified putative orthologous regions shared among lineages of the subfamily Cactoideae. Then we developed a probe set, named Cactaceae591, targeting both coding and noncoding nuclear regions for representatives from the subfamilies Pereskioideae, Opuntioideae, and Cactoideae. We also sampled inter- and intraspecific variation to evaluate the potential of this panel to be used in phylogeographic studies. We retrieved on average of 547 orthologous regions per sample. Targeting noncoding nuclear regions showed to be crucial to resolving inter- and intraspecific relationships. Cactaceae591 covers 13 orthologous genes shared with the Angiosperms353 kit and two plastid regions largely used in Cactaceae studies, enabling the phylogenies generated by our panel to be integrated with angiosperm and Cactaceae phylogenies, using these sequences. We highlighted the importance of using coalescent-based species tree approaches on the Cactaceae591 dataset to infer accurate phylogenetic trees in the presence of extensive incomplete lineage sorting in this family.

## 1. Introduction

Molecular phylogenetics has been a fundamental approach to understand the systematics and diversification of major plant groups, such as in the family Cactaceae [1]. The use of a few traditional molecular markers (e.g., plastid DNA regions) has been the primary source for phylogenetic and dating inference in Cactaceae [2]. However, the evolutionary relationship within tribes and genera remains largely inconclusive, partly due to the lack of phylogenetic signals in these molecular markers (see References [3,4,5,6,7] for examples). The poor phylogenetic resolution within Cactaceae is likely a reflection of the recent and rapid diversification experienced by the family since the late Miocene [8]. This tempo and mode of diversification may generate complex patterns of gene divergence, including large-scale incomplete lineage sorting (deep coalescence; see Reference [9]). Moreover, the reticulate evolution events experienced for some clades may also obscure the phylogenetic signals in recent radiation groups [10]. Accessing multiple independent loci allied to phylogenetic approaches that allow for gene tree heterogeneity [11] may be a better strategy to resolve challenging groups and recover highly supported phylogenies within this family.

For the past few decades, high-throughput sequencing techniques have become more affordable for non-model plants [12] and, more recently, have been applied to evolutionary studies in Cactaceae (see References [13,14,15]). Different sequencing strategies have fueled phylogenomic studies in cacti according to the research interest, including whole-genome (WGS) and transcriptome sequencing [14,16], reduced genome representation libraries (e.g., RAD-seq and GBS; see References [17,18]), and genome skimming for whole-plastome assembly [19,20,21,22]. In addition to allowing the test of evolutionary hypotheses in ways that were not previously feasible, these data represent an invaluable resource to survey for phylogenetically informative markers able to resolve contentious relationships at multiple levels of Cactaceae diversity. For instance, screening these genomic data for single-copy loci can provide a customized set of markers that is able to generate robust phylogenomic hypotheses when combined with hybridization-based target sequencing. Although all genomic strategies display pros and cons, especially regarding non-model species, the target-sequencing approach has intrinsic advantages. Among them, the most important are (i) the feasibility of capturing homologous single-copy genes across many individuals and species, thus decreasing the frequency of missing data and complicating downstream analyses; (ii) the requirement of a lower quality of DNA extraction, allowing for the use of herbarium specimens; and (iii) the more effective data integration with previous datasets [12].

Target sequencing by hybridization capture in plants is a cost-effective enrichment method for sequencing hundreds of putatively single-copy nuclear regions in multiple samples [23]. By screening the coding sequence variations in transcriptome data from related taxa, researchers can design sequence capture probes targeting orthologous coding sequences conserved across taxa. Furthermore, target sequencing allows us to capture specific single-copy genes of interest, such as those candidates to be under selection, which are accessed only casually, using non-targeted sequencing strategies. This method has become popular in angiosperm systematics and phylogenetic studies [24,25] and is largely fostered by the Angiosperms353 kit [26]. Acha and Majure [27] used Cactaceae genomic resources to develop a customized probe set for capturing coding sequences of 120 single-copy nuclear loci and complemented it with a subset of the Angiosperms353 probe set. Although universal kits such as Angiosperms353 have been successfully applied at both deep and shallow phylogenetic scales of many angiosperm families (see Reference [28]) and allow phylogenetic data integration among different taxa, lineage-specific probe sets are preferable in some cases. As an example, lineage-specific probe sets may be more effective in accessing relationships over multiple phylogenetic scales within rapidly radiating clades [29,30,31]. Furthermore, given the widespread occurrence of genomic duplications in angiosperms, lineage-specific kits are less prone to sample paralogous genes (but see Reference [32]), and this may affect the accuracy of phylogenetic inference.

Lineage-specific probe sets are generally designed to target conserved exon regions (see Reference [27]), while noncoding regions (i.e., introns and intergenic spacers) are generally sampled as off-target sequences (i.e., DNA sequences flanking the target exonic regions obtained by the Hyb-Seq approach [33]). Obtaining noncoding nuclear regions enables the accession of selectively unconstrained genomic variation that, allied with slow-evolving coding regions, may provide accurate phylogenetic inferences at multiple taxonomic scales [34,35,36], including rapid radiations [37]. Although noncoding regions have posted a huge impact on resolving shallow-level phylogenies [38,39], few studies have included them on customized probe sets (see References [40,41]).

Here, we present a newly developed probe set targeting both the coding and noncoding sequences of 591 putative orthologous regions for the subfamily Cactoideae, named Cactaceae591. We hypothesized that this probe set would be useful to perform phylogenetic studies across the phylogeographic–phylogenetic continuum of Cactaceae, as it includes regions with different levels of variation. We tested the informativeness of this panel sequencing 83 accessions for two datasets sampling: (1) at the family level on Cactaceae, including subfamilies Pereskioideae, Opuntioideae, and Cactoideae, with broad sampling of the tribe Cereeae; and (2) at the species level on Cactaceae, considering interspecific relationships and intraspecific variation within a group of closely related species of the genus *Cereus* Mill. [17]. We compared the performance of concatenated and coalescent-based phylogenetic reconstructions for both datasets. We highlighted the potential of Cactaeceae591 to produce well-resolved phylogenetic inferences at different taxonomic levels of the family Cactaceae. Furthermore, by overlapping 13 loci with Angiosperms353, the data from both probe sets may be partially aligned and integrated for phylogenetic purposes.

## 2. Materials and Methods

### 2.1. Target Loci Selection, Probe Design, and Library Preparation

We used Orthofinder v2.3.14 [42] to search for putative orthologous nuclear regions across 14 de novo transcriptomes and four genome assemblies available for Cactaceae in public repositories (References [14,16,43]; Supplementary Table S1). We constrained our search for orthologous nuclear regions shared among at least seven transcriptomes (at least one from the tribe Cereeae) and at least one of the four genome datasets selected in this study. We annotated the identified orthologous nuclear regions by using *Arabidopsis thaliana* (L.) Heynh. transcripts [44] and Caryophyllales representatives available on the PLAZA 5.0 dicots database [45], including an annotated cactus genome (*Selenicereus undatus* (Haw.), D.R.Hunt, designated as *Hylocereus undatus* by the authors [46]). The annotation was performed by using BLASTn with a minimum of 100 bp overlap and an e-value lower than 1 × 10^−15^ for *A. thaliana* transcripts. The annotation of Caryophyllales representatives considered metrics of a minimum identity of 85% and an e-value lower than 1 × 10^−80^, according to the genetic proximity across our samples and the Caryophyllales sequence taxa. We also obtained information about gene ontology from the PLAZA 5.0 dicots database (Supplementary Table S2). We removed cytoplasmic and contaminant (e.g., bacteria) sequences. After selecting the final putative orthologous nuclear regions on our dataset, we surveyed for shared target nuclear regions to those in the expanded version (mega353 target file [47]) of the Angiosperms353 kit [26], using BLASTn. We considered a match between our panel and the Angiosperm353 regions with an e-value lower than 1× 10^−10^, with a minimum of 50 bp overlap, and those that matched only one hit with the Angiosperm353 loci. Additionally, to enable Cactaceae591 datasets to be integrated with an array of previous Cactaceae phylogenetics studies (e.g., Reference [8]), we also targeted two plastid regions widely used in phylogenetic studies of Cactaceae in our probes: the protein-coding gene *rbcL* and the intergenic spacer *trnK-matK*.

The final dataset was composed of 591 orthologous target sequences, including coding and noncoding nuclear regions and two plastid regions. The noncoding regions are composed of the intronic, intergenic spacer, and anonymous (non-annotated) nuclear regions. The target regions identified in our survey ranged from 280 to 3590 bp. A set of 28,570 probes of 120 bp was designed by RAPiD Genomics LLC (Appendix A) with 3× tilling density. Approximately 58.1% of the probes were designed on exons, 32.27% on intronic and intergenic regions, and 9.63% on anonymous regions. A lower probe density was designed for plastid *rbcL* and *trnK-matK* regions to avoid overrepresentation of these regions in the dataset.

### 2.2. Sampling, DNA Extraction, and Library Preparation

We designed our sampling to evaluate the performance of Cactaceae591 at the family and species levels (for more details, see Supplementary Table S3). The family-level dataset (hereafter, the Cactaceae dataset) was generated by sampling 57 species from the subfamilies Pereskioideae, Opuntioideae, and Cactoideae, with an emphasis on the tribe Cereeae (sensu [48]). This sampling covers divergence events spanning from the Oligocene (~26.9 MyA) to Pleistocene (~2.5 MyA, [8,49,50,51]). A species-level dataset (hereafter, the Cereus dataset) was generated by sampling 27 representatives of eight species and two subspecies that compose a monophyletic group (clade A) within the genus *Cereus*, as well as three species from sister clades (*Cereus spegazzini*, *Cereus mirabella*, and *Cereus kroenleinii*), used as outgroups (see Reference [17]; Supplementary Table S3). Clade A is composed of closely related species distributed in the Dry Diagonal of South America (Cerrado, Caatinga, and Chaco biomes) that diverged from early to late Pleistocene (2.1 Mya to 80 kya, [51]). We sampled a single individual from multiple populations for most species of this clade (see Supplementary Table S3 for more details).

DNA extraction was performed by using 80 to 100 mg of root or epidermis grounded with steel beads on TissueLyser II (Qiagen, Hilden, Alemanha). We performed DNA extraction from fresh and −80 °C preserved tissues and from herbarium material (see Supplementary Table S3 for more details). Genomic DNA was extracted by using a high-salt CTAB protocol slightly modified from Inglis et al. [52]. DNA integrity, quality, and quantity were checked by using 1% agarose gel Nanodrop absorbance ratios and Qubit dsDNA High Sensitivity (Thermo Scientific, Waltham, MA, USA), respectively.

Library preparation was performed by RAPiD Genomics for Illumina sequencing, utilizing their high-throughput workflow with proprietary chemistry. Briefly, DNA was sheared to a mean fragment length of 400 bp, and fragments were end-repaired, followed by incorporation of unique dual-indexed Illumina adaptors and PCR enrichment. Sequence capture was performed by using RAPiD Genomics proprietary chemistry and workflows. Briefly, fully constructed libraries were hybridized to 120 bp probes, and probe/DNA hybrids were captured on streptavidin beads, washed, and PCR amplified. Samples were pooled equimolar and sequenced on a NovaSeq 6000 (2 × 150 bp).

### 2.3. Sequencing Assembly, Alignment, and Genetic Variability of Target Loci

The raw reads were trimmed by using AdapterRemoval v2 [53], removing poor-quality reads (phred < 20) and reads smaller than 60 bp. We assembled and mapped the trimmed reads by using HybPiper pipeline v1.3 [54] across the 591 target regions. To enable efficient locus recovery, we constructed a variable reference sequence file by using sequences from multiple species across subfamily Cactoideae (Appendix B). The trimmed reads were mapped to reference sequences, using BWA [55], and then assembled into contigs with a minimum coverage of 8×, using SPADES [56]. Summary statistics of mapped reads and recovered target regions were obtained by using hybpiper_stats.py.

We detected putative paralogous regions in our dataset by using the command paralog_investigator.py on the HybPiper pipeline. Putative paralog regions were checked on gene trees inferred with IQ-TREE 1.6 [57] and by using the contigs from each sample obtained, using the paralog_retriever.py script on HybPiper. Putative paralogs shared among more than 10% of samples were removed from the final dataset. We also excluded regions with more than 70% missing data on the family-level sample.

The final dataset was automatically aligned by using MAFFT v7 [58]. Indel-rich regions were trimmed by using trimAL v1.3 [59] with the function –gt 0.7. The alignments were concatenated by using AMAS v0.94 [60]. Summary statistics (alignment length, number, and variable sites and parsimony-informative sites) were calculated by using AMAS v0.94 [60] for our two main datasets (Cactaceae and Cereus datasets).

We also explored nuclear regions shared between the Cactaceae591 and Angiosperm353 probe sets, following a similar strategy described by Siniscalchi et al. [32]. Our aim here was to test whether representatives from the same taxon and sequenced by different probe sets could be recovered as monophyletic groups. We selected representatives of major clades of Cactaceae sequenced with our probe set (obtained in this study) and with the Angiosperm353 probe set obtained by Baker et al. [25] (see Supplementary Table S4 for more details about this sampling). We assembled this dataset by using a reference sequence file of the expanded version (mega353 target file [47]) of the Angiosperms353 kit [26], implementing the pipeline of Hybpiper, as described above. The loci recovered in more than 50% of the selected samples were aligned by using MAFFT v7 [58], and indel-rich regions were trimmed by using trimAL v1.3 [59] with the function –gt 0.5. The final concatenated alignment obtained with AMAS v0.94 was used as input to infer a maximum likelihood tree on IQ-TREE 1.6 [57] with 10,000 ultrafast bootstrap (BS) replicates [61].

### 2.4. Phylogenetic Inference

To investigate the efficiency of Cactaceae591 coding and noncoding sequences separately for phylogenetic reconstructions in Cactaceae, we explored different data matrices within each dataset (Supplementary Table S3). For the Cactaceae dataset, we explored the phylogenetic signal, focusing only on coding regions (exonic sequences; Cactaceae-coding sub-dataset, herein) and all target nuclear regions (exons, introns, intergenic spacers, and anonymous nuclear regions; Cactaceae-all sub-dataset, herein). For the Cereus dataset, we explored a matrix including only noncoding nuclear regions (introns, intergenic spacers, and anonymous nuclear regions; Cereus-noncoding sub-dataset) and a matrix with all target nuclear regions (Cereus-all sub-dataset). Our main focus was to evaluate the phylogenetic signal of orthologous nuclear regions under different levels of selective constraints. We did not include the plastidial regions in our phylogenetic analysis, as the value of these regions for phylogenetic purposes was already tested in previous studies.

We performed phylogenetic inference by using both concatenated and coalescent approaches. Maximum likelihood (ML) trees were generated by using a concatenated matrix in IQ-TREE 1.6 [57] with 10,000 ultrafast bootstrap (BS) replicates [61], applying the -bb option. We assumed the best-fit model for each partition by using ModelFinder [62], which is available on IQ-TREE. The ML trees of Cactaceae were rooted by using *Pereskia bahiensis* Gürke *and Leunbergeria aureiflora* (F. Ritter) Lodé, while *Cereus* clade A gene trees were rooted by using *C. mirabella* N.P. Taylor and *C. kroenleinii* N.P. Taylor. Branch support of ML trees (BS) was classified into three categories: high (>95), moderate (95–70), and low (<70).

The coalescent species trees were inferred by a summary approach on ASTRAL-III v5.6 [63], using ML gene trees generated in IQ-TREE v1.6 [57], with 10,000 ultrafast bootstrap replicates [61]. Nodes in gene trees with BS <50 were collapsed. The branch supports of species trees were accessed by local posterior probabilities (LPPs), which were computed based on gene tree quartet frequencies [64]. Branch support of coalescent-based trees (LPP) was classified as high support (>0.95), moderate support (0.95–0.70), and low support (<0.70).

## 3. Results

### 3.1. Sequencing and Datasets

Approximately 96 million reads were obtained for all samples after the trimming process, averaging 1.1 million reads per sample. The mapped reads corresponded to 72.5 ± 4.2% (mean ± SD) of the total trimmed reads (Supplementary Table S5). The total target regions recovered from each sample ranged from 417 to 566 (Supplementary Table S5). The two plastid regions included in our orthologous probe set were recovered for all samples. Most of the target regions (547) were captured for more than 70% of our sampling, with slightly more regions recovered on the subfamily Cactoideae (550) compared to the subfamilies Pereskioideae (517) and Opuntioideae (429) (Supplementary Figure S1). We identified an average of 12 putative paralogs per sample (Supplementary Table S4), and 45 paralogous regions were shared with approximately 10% of our sampling. Considering the target loci identified as paralogs and loci that failed for most samples, we excluded 74 target regions from our datasets. We identified 13 target nuclear regions shared between the Cactaceae591 and Angiosperm353 probe sets according to the BLAST results (Supplementary Table S2), but only ten were shared by more than one representative sequenced with the Cactaceae591 probe set (Supplementary Table S5). The sequences overlapping in these loci varied from 75 to 666 base pairs. The phylogenetic inference recovered the monophyly of representatives of major clades of Cactaceae from different sequencing strategies (Cactaceae591 vs. Angiosperm353; Supplementary Figure S2), revealing the integration of ten target nuclear loci sequenced by the Cactaceae591 and Angiosperm353 panels.

The final matrix consisted of 512 orthologous regions containing 292 exonic, 328 intronic, and 67 anonymous sequences. These sequences resulted in a matrix of 748,848 bp with 194,892 parsimony informative sites (PIS) for the Cactaceae dataset and 864,313 bp with 23,602 PIS for the Cereus dataset (Table 1). Anonymous regions showed a higher percentage of missing data but also presented a higher number of PIS than exonic regions (Table 1).

### 3.2. Phylogenetic Reconstructions

**Figure 1:**
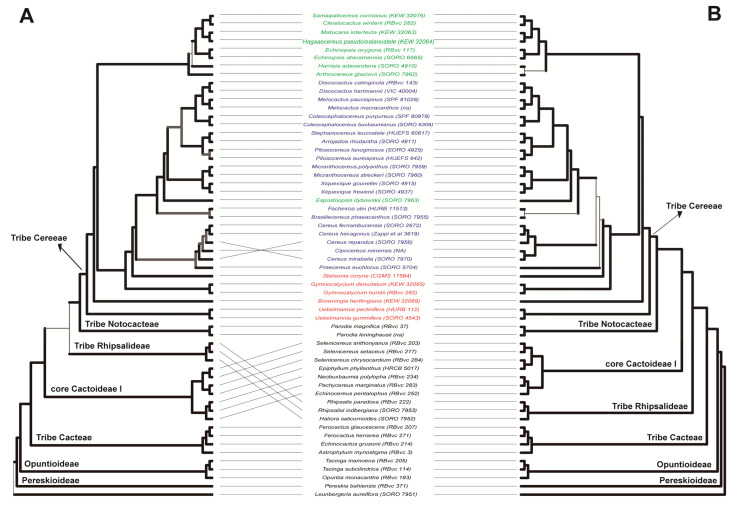
Comparison of coalescent-based species trees of Cactaceae estimated with Cactaceae-coding (**A**) and Cactaceae-all (**B**) sub-datasets. Cactaceae-coding and Cactaceae-all sub-datasets included 290 and 512 nuclear regions, respectively. The branches with high support (LPP > 0.95) are colored with thick dark lines, the branches with moderate support (LPP 0.95–0.70) are colored with thick gray lines, and the branches with low support (LPP < 0.70) are shown as thin light lines. Voucher number of each accession is provided in parenthesis after species names. Species from the subtribes Rebutinae, Trichocereinae, and Cereinae are colored red, green, and blue, respectively (sensu Nyffeler and Eggli [48]). LPP: local posterior probability.

Phylogenetic inferences recovered a family-level tree congruent with the current Cactaceae systematics (Figure 1). The subfamilies and the tribes sampled were recovered as monophyletic groups, and the phylogenetic relationships were generally congruent with those inferred in previous studies [5,8,27,49]. Furthermore, most of the Cactaceae deep nodes were recovered with high support, regardless of the sub-dataset and inference method used (Figure 1 and Supplementary Figure S3). A topological incongruence was observed between phylogenetic reconstruction approaches. The coalescent-based inference recovered *L. aureiflora* as the sister of all remaining Cactaceae, followed by *P. bahiensis* (Figure 1), while the ML inference grouped both species in the same branch (Supplementary Figure S3). An additional major incongruence was observed among sub-datasets involving the position of Rhipsalideae in relation to core Cactoideae I and II. Using the Cactaceae-coding sub-dataset, the representatives of this tribe were clustered in a polytomic node with the highly supported clades of core Cactoideae I and II (Figure 1 and Supplementary Figure S3). However, by using the Cactaceae-all sub-dataset, we resolved these relationships with high support, setting the tribe Rhipsalideae as the sister of a clade clustering core, Cactoideae I and II (Figure 1 and Supplementary Figure S3).

Some shallow-level incongruences were observed between Cactaceae sub-datasets involving the relationships among the *Praecereus*, *Cipocereus*, and *Cereus* genera (Figure 1 and Supplementary Figure S3). For example, *Cipocereus* was highly supported within the clade of *Cereus* species in all inferences. A further example of shallow-level incongruence involved *Praecereus*, which was allocated as a sister clade of *Cereus* species, depending on the dataset and phylogenetic approach used (Figure 1 and Supplementary Figure S3).

**Figure 2:**
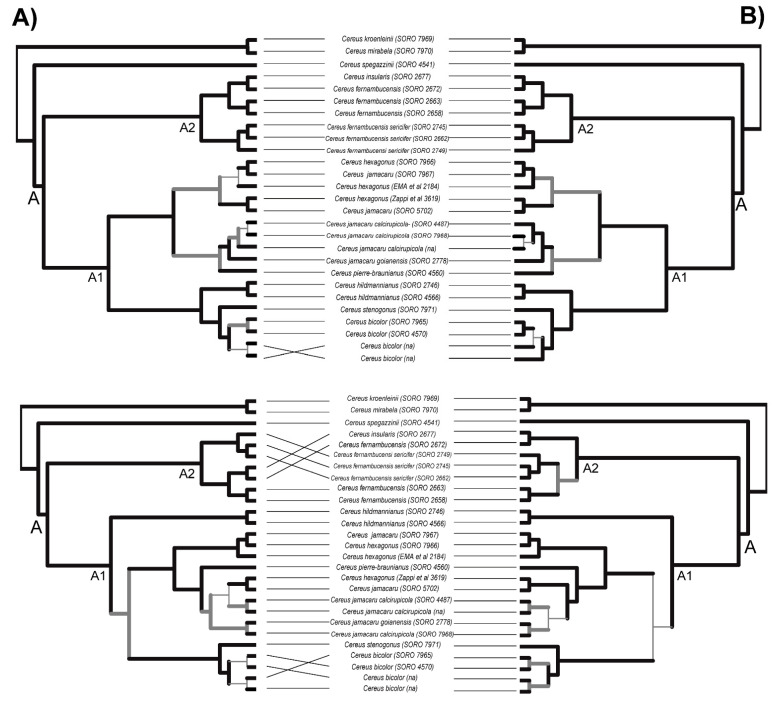
Comparison of phylogenies of Cereus clade A inferred with Cereus-noncoding (**A**) and Cereus-all (**B**) sub-datasets, using coalescent-based (upper) and ML concatenated (down) approaches. The branches with high support (LPP > 0.95 and BS > 95) are highlighted with thick branch lines, the branches with moderate support (LPP 0.95–0.70 and BS 95–70) are colored with thick gray lines, and the branches with low support (LPP < 0.70 and BS < 70) are shown as thin light lines. LPP, local posterior probability; BS, ultrafast bootstrap. Voucher number of each accession is provided in parenthesis after species names.

The phylogenetic backbone for *Cereus* clade A recovered the same relationships estimated for this clade by using RAD-Seq data with high branch support (Figure 2; see Reference [17]). Furthermore, all conspecific samples nested together, with the exception of the representatives of *C. hexagonus* and *C. jamacaru* (Figure 2). The hypothesis of a peripatric origin of *C. insularis* from coastal populations of *C. fernambucensis* subsp. *fernambucensis* in Northeastern Brazil [51,65] was corroborated with all sub-datasets and phylogenetics methods. Only the ML tree inferred with the Cereus-all sub-dataset provided a slightly different relationship among these taxa, nesting *C. fernambucensis* subsp. *sericifer* within the clade of *C. fernambucensis* subsp. *fernambucensis*.

We observed that, in general, the use of sub-datasets, including all target regions (both coding and noncoding), resulted in an increase in branch support to resolve relationships within both Cactaceae and Cereus clade A groups (Table 2).

## 4. Discussion

### 4.1. Cactaceae591: A Lineage-Specific Probe Set to Perform Evolutionary Studies in Cactus

Here, we describe Cactaceae591, a new lineage-specific probe set targeting both coding and noncoding orthologous regions in Cactaceae. Based on the large number of polymorphic sites recovered, the presence of gene regions with different levels of nucleotide variation, and the robustness of our phylogenetic inferences at both the family and species levels, we highlight the relevance of this probe set at multiple taxonomic levels within Cactaceae.

Previous evolutionary studies on recent and rapid divergent groups of angiosperms reported a robust and accurate phylogenetic reconstruction when adding information from noncoding nuclear regions [13,37,38,39,40]. Nevertheless, few target sequencing kits incorporated probes designed to capture intronic and intergenic regions (see References [40,41]). By integrating coding and noncoding sequences that are expected to exhibit different evolutionary rates, Cactaceae591 approaches the ideal characteristics of molecular markers for resolving relationships in taxa that have undergone recent radiation [66]. The increase of branch support when using both coding and noncoding data highlights the value of combining slow- and fast-evolving sequences in our phylogenetic inferences, especially at the species level. We acknowledge that our intraspecific sampling was limited to one sample per population, preventing us from testing the capability of Cactaceae591 to recover variation within populations. However, taking into account the intraspecific variation recovered in our study, we believe that the noncoding regions available in Cactaceae591 are also promising for phylogeographic and population studies on the family.

Overall, the trees inferred from the Cactaceae dataset were congruent with previous phylogenetic studies, regardless of the phylogenetic approaches (concatenation or coalescent-based reconstructions) or sub-datasets used (coding and noncoding regions). The major discordances occurred on terminal nodes and/or on short deep internodes, as is commonly seen on cactus lineages, due to the rapid and recent divergence experienced by many clades in this plant family [1,17]. It is important to highlight the importance of the implementation of coalescent-based phylogenetic methods in cacti, as there is extensive incomplete lineage sorting generating gene tree heterogeneity in this family [14], a scenario in which the concatenation approach can lead to strongly supported misleading relationships [11].

The results generated with Cactaceae591 have the potential to be integrated with phylogenomic data that are available from other studies, as Cactaceae591 shares thirteen orthologous regions with the Angiosperm353 kit, as well as two plastid markers commonly used in phylogenetic and phylogeographic studies in cacti [2]. Recent studies have successfully used this universal probe set on Cactaceae [25,27]. However, by targeting both orthologous coding and noncoding nuclear regions, Cactaceae591 has greater potential and flexibility to infer phylogenetic relationships at both the shallow and deep levels of the evolution of Cactaceae. Hence, the combination of lineage-specific probe sets with universal panels may benefit both the cacti- and plant-specialist communities [32,67,68].

### 4.2. Notes on the Phylogenetic Relationships

Although our main focus was to describe Cactaceae591, some of our phylogenetic results are worth noting. Overall, the backbone phylogeny was consistent across different methods and sub-datasets, recovering the monophyly of tribes and genera of the subfamily Cactoideae. The sister relationship of *L. aureiflora* and the remaining Cactaceae taxa, followed by *P. bahiensis*, agrees with the paraphyly hypothesis for *Pereskia* s.l. [15,69] and with those inferred by using the Cactaceae120 probe set [27]. Phylogenetic inferences based on plastid DNA [4,5,49] have grouped the tribe Rhipsalideae as a member of Core Cactoideae II. Our results using all target nuclear regions resolved this tribe with high support as sister of the clade clustering core Cactoideae I and II, while using only coding nuclear regions; Rhipsalideae assumes a polytomy with these clades. Interestingly, an inconsistent relationship of Rhipsalideae with core Cactoideae was also observed by Acha and Majure [27], using the Cactaceae120 dataset. Future studies using a wider sampling of the representatives of the subfamily Cactoideae are likely required to elucidate these contentious relationships with high accuracy.

Our main sampling focused on the tribe Cereeae and its subtribes. Beyond the aware paraphyletic circumscription of subtribe Rebutinae [48], the monophyly of subtribes Cereinae and Trichocereinae is still under debate (see Reference [70]). Considering the circumscription proposed by Nyffeler and Eggli [48] for the tribe Cereeae, our results recovered the subtribe Trichocereinae as polyphyletic and the subtribes Cereinae and Rebutinae as paraphyletic groups. For instance, *Espostoopsis dybowskii* was recovered within the Cereinae clade, although this taxon is traditionally considered a member of the subtribe Trichocereinae. In the phylogenies of Ritz et al. [3], Schlumpberger and Renner [71], Lendel et al. [72], and Bombonato et al. [17], it is also possible to observe the members of these subtribes as nonmonophyletic taxa. Finally, our results found the same contentious relationships involving the genera *Cereus*, *Cipocereus*, and *Praecereus* inferred with plastid [50] and RAD-Seq data [17].

The target regions selected here were useful for distinguishing relationships within closely related species of the genus *Cereus*. The relationship within this clade consistently agreed with previous phylogenomic results [17] when applying the Cereus-all sub-dataset allied with coalescent-based inferences. The inability to completely separate the currently recognized taxonomic species of subclade *C. jamacaru* is a problem not only associated with rapid diversification of the group but also possibly with a misleading taxonomy of this genus (see Reference [73]). The potential to resolve inter- and intraspecific relationships demonstrated by this probe set may also be helpful for species delimitation purposes within this group.

## 5. Conclusions

The Cactaceae591 probe set showed to be a powerful strategy to resolve phylogenetic relationships at deep and shallow taxonomic levels, displaying topological congruences with previous phylogenetic studies. In addition, by targeting noncoding fast-evolving regions, Cactaceae591 potentially captures neutral variation that is useful for phylogeographic studies. The sharing of thirteen loci with the Angiosperms353 kit allows for the integration of the Cactaceae591 dataset across angiosperm phylogenetic studies.

## Figures and Tables

**Table 1:** Summary statistics of nucleotide variation calculated for coding, noncoding, and anonymous regions in the Cactaceae and Cereus datasets. Note: bp, base pairs; S, variable sites; PIS (%), parsimony informative sites (proportion of PIS).

Dataset	Alignment Length (pb)	% Missing Data	S	PIS (%)
Cactaceae				
Exons	258,375	12.83	100,715	54,156 (0.211)
Introns	309,041	23.28	149,244	78,597 (0.254)
anonymous regions	154,174	64.45	44,159	22,416 (0.145)
Cactaceae-coding	258,375	12.83	100,715	54,156 (0.211)
Cactaceae-all	721,59	33.52	294,118	155,169 (0.215)
*Cereus* Clade A				
Exons	303,688	12.67	24,729	10,278 (0.034)
Introns	424,012	5.49	42,732	18,454 (0.044)
anonymous regions	136,613	25.02	11,036	5148 (0.038)
Cereus-noncoding	560,025	15.26	53,768	23,603 (0.042)
Cereus-all	864,313	14.39	78,497	33,880 (0.039)

**Table 2:** Node support in the phylogenetic trees inferred from the Cactaceae and Cereus datasets, using different sub-datasets (coding, noncoding, and all sequences) and phylogenetic approaches. Branch support values of coalescent (LPP) and concatenated (BS) trees were classified as high (>0.95, >95), moderate (0.95–0.70, 95–70), or low (<0.70, <70), respectively.

Dataset	Total	High Support	Moderate Support	Low Support
Cactaceae-coding				
concatenated	57	51	5	1
coalescent-based	57	48	7	2
Cactaceae-all				
concatenated	57	52	3	2
coalescent-based	57	53	0	4
Cereus-noncoding				
concatenated	26	19	4	3
coalescent-based	26	19	4	3
Cereus-all				
concatenated	26	19	5	2
coalescent-based	26	21	3	2

## Data Availability

The data presented in this study are available in the Supplementary Materials. Accession numbers for all sequence data used to design the sequence capture array are presented in Table S1. The raw reads of target sequence capture data are available on the National Center for Biotechnology Information Sequence Read Archive (NCBI SRA), under the BioProject accession number PRJNA812417.

## Supplementary Materials

The supporting information can be downloaded at https://www.mdpi.com/article/10.3390/genes13040707/s1. Figure S1: Heatmap indicating 591 orthologs’ recovery success per sample; scale color indicates success rate. Figure S2: Coalescent species trees estimated with ten loci shared by Cactaceae591 and Angiosperm353 probe sets. The BS values are displayed on the nodes. Species with suffix (591) were sequenced with a Cactaceae591 kit, and those with (353) were sequenced with an Angiosperm353 kit. The branches with high support (BS > 95) are colored with thick branch lines, the branches with moderate support (BS 95–70) are colored with thick gray lines, and the branches with low support (BS < 70 on ML inferences) are shown as thin light lines. Figure S3: Maximum likelihood phylogenies inferred from Cactaceae-coding (A) and Cactaceae-coding (B) datasets. The branches with high support (BS > 95) are colored with thick branch lines, the branches with moderate support (BS 95–70) are colored with thick gray lines, and the branches with low support (BS < 70 on ML inferences) are shown as thin light lines. Species colored red are from the subtribe Rebutinae, green are from the subtribe Trichocereinae, and blue are from the subtribe Cereinae (sensu Nyffeler and Eggli [48]). Table S1: Genomic data of representatives from subfamily Cactoideae used for identification of orthologous genes. Table S2: Details about the 591 orthologous regions selected in the present study, including probe number, annotation according to *Arabidopsis thaliana* and the PLAZA database, and paralog assignment for each gene. Table S3: Sampling information of Cactaceae species used in this study. RBvc: Cactário do Jardim Botânico do Rio de Janeiro; SORO: Herbário do Centro de Ciências e Tecnologias para a Sustentabilidade; HURB: Herbário do Recôncavo da Bahia; HUEFS: Herbario da Universidade Estadual de Feira de Santana; KEW: Kew DNA bank; SPF: Herbário da Universidade de São Paulo; VIC: Herbário da Universidade Federal de Viçosa. CGMS: Herbário da Fundação Universidade Federal de Mato Grosso do Sul; HRCB: Herbário Rioclarense; na: not available. Table S4: Sampling information used in analysis of shared nuclear regions with Cactaceae591 and Angiosperm353 probe set. Table S5: Summary statistics of locus recovery analysis performed on HybPiper for the sampling sequenced and mapped with Cactaceae591 probe set (see Table S3). Table S6: Length recovery of locus shared by both Cactaceae591 and Angiosperm353 probe sets obtained by HybPiper. The numbers of each column represent the locus name of the Angiosperm353 panel. We selected representatives from major clades of Cactaceae sequenced with the Cactaceae591 probe set (this study) and with the Angiosperm353 probe set (Baker et al. 2021), including representatives from closely related families of Cactaceae.

## Appendix A

Probe set sequences used to capture 591 target orthologous regions of Cactaceae to construct the target enrichment library.

## Appendix B

Reference sequences for the 591 target orthologous regions identified from genomic and transcriptome resources detailed in Supplementary Table S1.
