# Supplementary material for: A target Capture Probe Set Useful for Deep- and Shallow-Level Phylogenetic Studies in Cactaceae
Source: Genes (Basel). 2022 Apr 17;13(4):707. doi: 10.3390/genes13040707 (PMC9032687; doi:10.3390/genes13040707)
Supplement: Supplementary file 1 [file genes-13-00707-s001.zip › Fig S1.pdf]

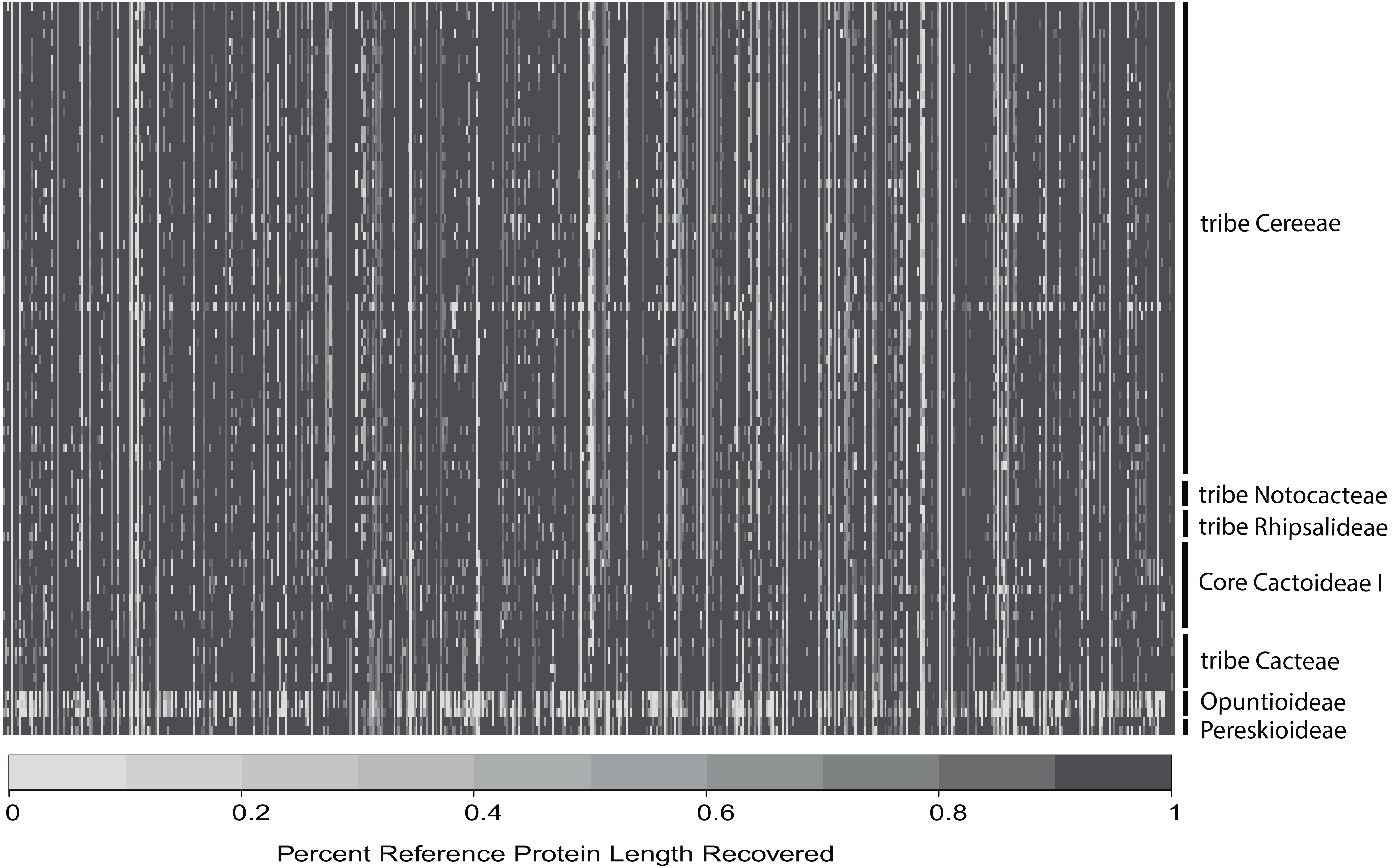
tribe Cereeae
tribe Notocacteae
tribe Rhipsalideae
Core Cactoideae I
tribe Cacteae
Opuntioideae
Pereskioideae
0
0.2
0.4
0.6
0.8
1
Percent Reference Protein Length Recovered
