# Supplementary figures and images for: A target Capture Probe Set Useful for Deep- and Shallow-Level Phylogenetic Studies in Cactaceae
Source: Genes (Basel). 2022 Apr 17;13(4):707. doi: 10.3390/genes13040707 (PMC9032687; doi:10.3390/genes13040707)

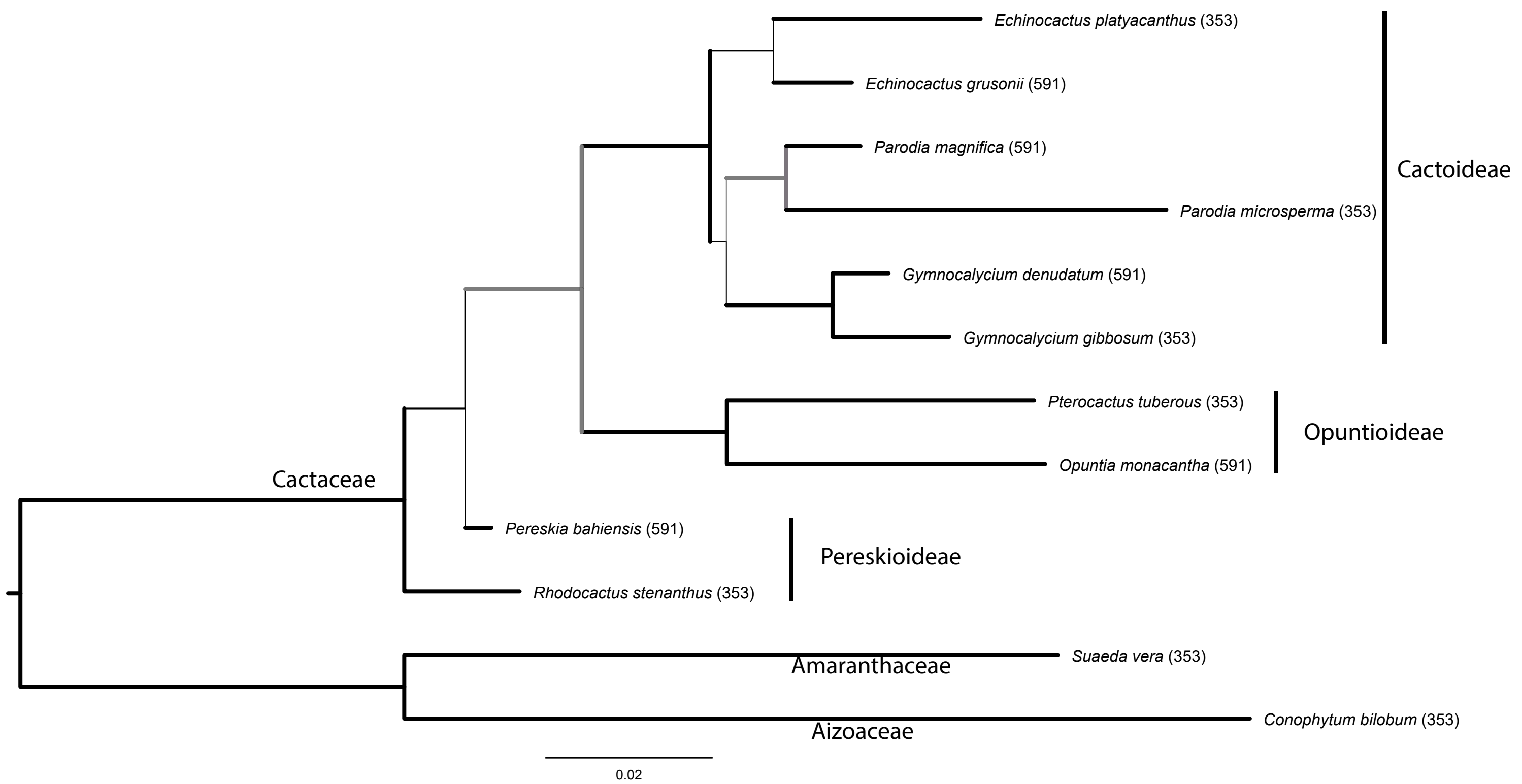

Supplement: Supplementary file 1 [file genes-13-00707-s001.zip › Fig S2.pdf]

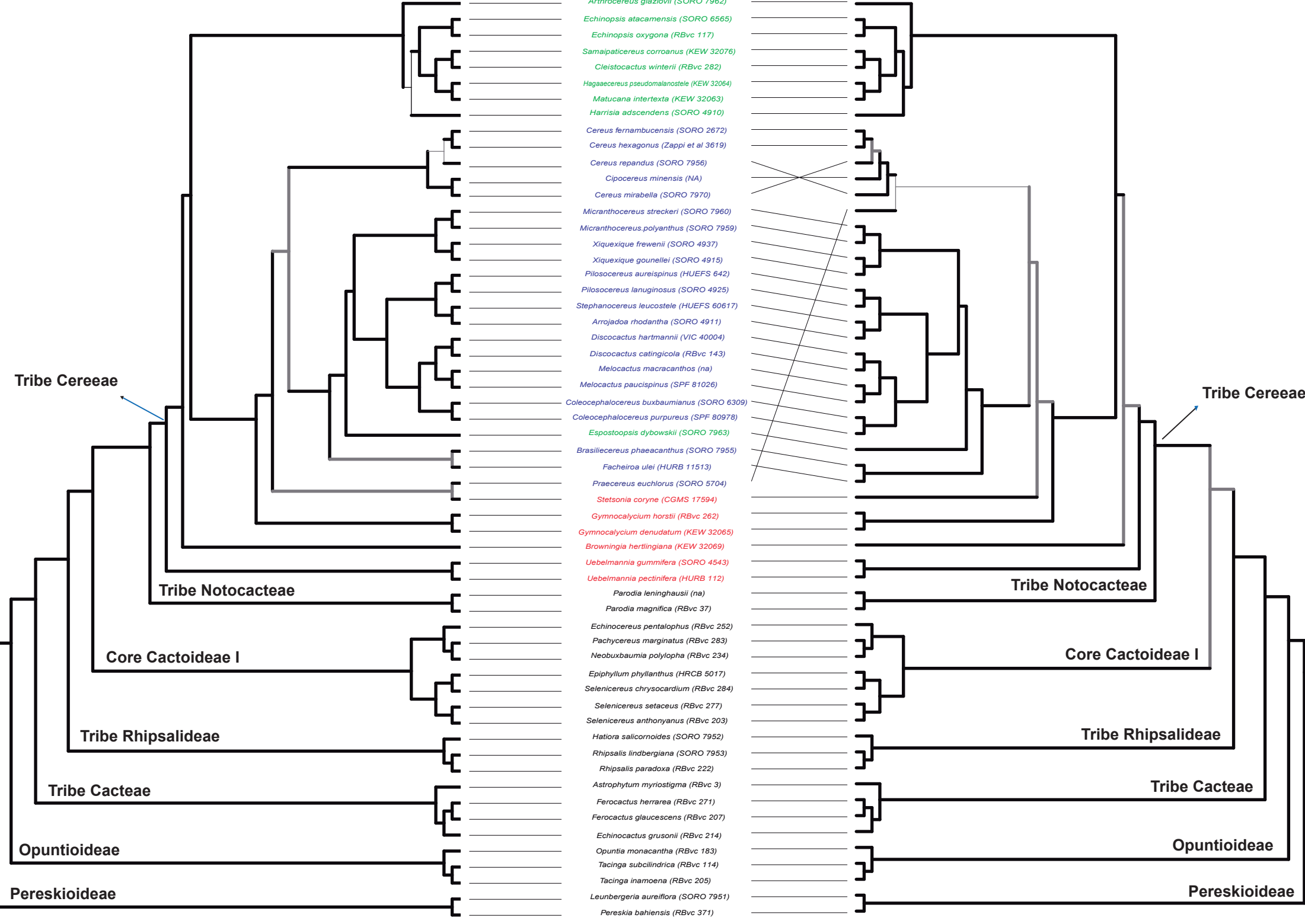

Supplement: Supplementary file 1 [file genes-13-00707-s001.zip › Fig S3.pdf]
